# Vocal complexity influences female responses to gelada male calls

**DOI:** 10.1038/srep19680

**Published:** 2016-01-21

**Authors:** Morgan L. Gustison, Thore J. Bergman

**Affiliations:** 1 Department of Psychology, University of Michigan, 530 Church Street, Ann Arbor, MI, 48109, USA; 2 Department of Ecology and Evolutionary Biology, University of Michigan, 830 North University, Ann Arbor, MI, 48109, USA

## Abstract

Extensive research indicates that inter-sexual selection drives the evolution of complex vocal communication in birds, but parallel lines of evidence are almost entirely absent in mammals. This dearth of evidence, particularly among primates, limits our understanding of the link between sociality and vocal complexity. Here, we use a playback experiment to quantify how wild female geladas (*Theropithecus gelada*) respond to three call types that are ‘derived’ (i.e., unique to geladas) and made by males during various affiliative contexts. These derived calls appeared to be highly salient and preferable to females: they looked longer towards and spent more time in proximity to playbacks of male vocal sequences containing one of the derived calls than to sequences containing only common and less elaborate ‘grunt’ calls. Our results provide the first experimental evidence for vocal elaboration as a male-specific strategy to maintain social bonds with females in non-human primates.

Human’s ability to combine sounds together into an endless array of meaningful words and sentences is unique, making the evolutionary roots of language a focus of intense interest^1^,^2^. Despite this uniqueness, several aspects of language can be studied comparatively^3^,^4^. In particular, there is tremendous interest in documenting the diversity of ‘vocal complexity’ in animals^5^. Vocal complexity is typically defined as the number of different vocalizations a species can make, or vocal repertoire size, and this trait differs extensively across taxa^6^,^7^,^8^. This diversity is useful because it allows for comparative studies that can identify the main types of selective pressures driving the evolution of complex forms of communication. Several comparative studies in birds and mammals, including humans, suggest an important role for social pressures in the evolution of vocal complexity by showing that broad measures of vocal complexity (e.g., repertoire size) are positively associated with sociality (e.g., group size)^6^,^7^,^9^,^10^,^11^,^12^. However, our understanding of the specific social functions of individual features of vocal systems, such as complex strings of sound, is more limited.

The most comprehensive data on the social functions of complex strings of sound come from research on inter-sexual selection in bird song^13^,^14^,^15^. Male songbirds (Passeriformes) often produce songs during courtship, and females show preference for males with larger repertoires of syllable, phrase, or song types^16^,^17^,^18^,^19^,^20^,^21^ and males producing songs composed of more complex elements^14^,^22^,^23^. In either case, sexual selection of songbird vocal complexity appears to act at the level of the “sequence”, meaning that the functional unit of sound is the combination of elements rather than the individual elements themselves. While individual elements can affect responses to the song, each song element has a similar shared function (e.g., mate attraction). In some species, females may gain direct or indirect benefits by using vocal complexity as an index of male quality^24^,^25^, while for other species, vocal complexity may primarily be a way for males to exploit females’ auditory sensory biases^19^. Regardless of the specific pathway leading to a preference, the outcome is the same: in many bird species, vocal complexity can facilitate inter-sexual social interactions. However, we do not know if these findings are unique to songbirds and their unusual vocal system. The function of complexity in other animal vocal systems remains largely unexplored.

Most relevant for understanding the origins of language are the vocal systems of primates. Humans and other primates exhibit several homologies in the brain circuitry involved with communication^26^, and like humans, many primates maintain long-term relationships and live in large social groups^27^. These shared traits make primates useful comparative models to explore the role that sociality plays in the evolution of vocal complexity. Yet, unlike songbirds, non-human primates exhibit relatively small repertoires of discrete and graded sounds (i.e., calls), and much less is known about how and why they combine multiple call types into sequences^4^. Broad comparative research suggest that primate species with large vocal repertoires are characterized by living in large social groups and spending a great deal of time engaged in affiliative behaviors^12^. Moreover, narrower comparative studies show that some non-human primate taxa exhibit a greater degree of vocal complexity than their close relatives because they produce more types of calls during within-group aggressive or affiliative social interactions^9^,^28^. Together, this body of comparative work suggests that some primates have more complex vocal systems because they produce unique ‘derived’ call types that evolved to better facilitate social interactions. For example, mandrills (*Mandrillus sphinx*) produce a unique suite of long distance contact calls that are thought to play a species-specific role in coordinating group movements in densely forested environments; analogous call types are not found in closely related species like baboons and geladas^29^.

Identifying derived call types and the social contexts in which they are produced are the first steps towards understanding how vocal complexity functions in primates. We also need complimentary studies that examine how these derived call types are perceived by potential receivers, which we currently know little about in primates and other mammals^30^,^31^,^32^. We address this gap by investigating behavioral responses to derived calls in wild geladas (*Theropithecus gelada*), a primate known for its large and unique vocal repertoire and for which derived call types (i.e., calls with no clear analogs in the vocal repertoires of their close baboon relatives) have already been identified^28^,^33^,^34^,^35^,^36^. Three of these derived call types – “moans”, “wobbles”, and vocalized “yawns” – are of particular interest because they are produced almost exclusively by adult males and are the most acoustically elaborate of the derived calls. Moans are long in duration, wobbles have a high degree of frequency modulation, and yawns take up a large frequency bandwidth^28^,^34^. Due to the male-biased production and their elaborate form, it appears that these calls are sexually selected call types, although this possibility has never been experimentally tested.

Gelada males typically produce moans, wobbles, and yawns in vocal sequences, and they do this by combining them with a homologous call type – exhaled grunts – and another ‘derived’ call type – inhaled grunts – both of which are commonly produced by both male and female geladas^36^. Males produce grunt sequences containing no, one, or multiple elaborate derived calls during close-range affiliative social interactions with females (e.g., approaches, allogrooming, and after female-female conflicts)^28^,^33^,^36^. As in birds, geladas combine different types of sounds in a single social context, suggesting that the function of each element is to add to the sequence complexity rather than to serve a unique social function. However, it remains unknown whether the vocal sequences containing elaborate derived calls and grunts elicit different responses from those containing only grunts. One intriguing possibility is that, as in birdsong, the diversification of gelada males’ affiliative vocal sequences may function to attract or bond with their female counterparts^28^. Geladas aggregate into extremely large groups of over 1000 individuals that are made up of smaller ‘harem-like’ reproductive units composed of a dominant leader male, up to a few subordinate follower males, and several females and their dependent offspring^37^. Leader males of reproductive units that use effective strategies to maintain their long-term social relationships may decrease the chance of being cuckolded by within-unit subordinate males^38^,^39^ or out-competed by a non-unit ‘bachelor’^40^. It is still unknown in geladas, and in primates more generally, whether vocal sequences containing elaborate derived calls from males influence female behavior in a way that could benefit male fitness. Such a finding would be the first evidence for inter-sexual selection of vocal complexity in a non-human primate.

We build off prior studies on the production of vocal complexity in male geladas by examining the perception of vocal complexity by female geladas. Using an experimental playback design, we modeled established behavioral assays to assess female responses to male vocal displays^41^,^42^,^43^,^44^. First, we tested whether or not female geladas discriminate between vocal sequences that do or do not contain one of the elaborate derived calls and grunts by comparing females’ visual orientation towards simulated male vocal sequences. Second, we tested whether females show a ‘preference’ for these derived calls by comparing the amount of time that females spent in proximity to simulated derived call and grunt only sequences.

## Results

### Visual orientation towards the speaker

**Figure 1:**
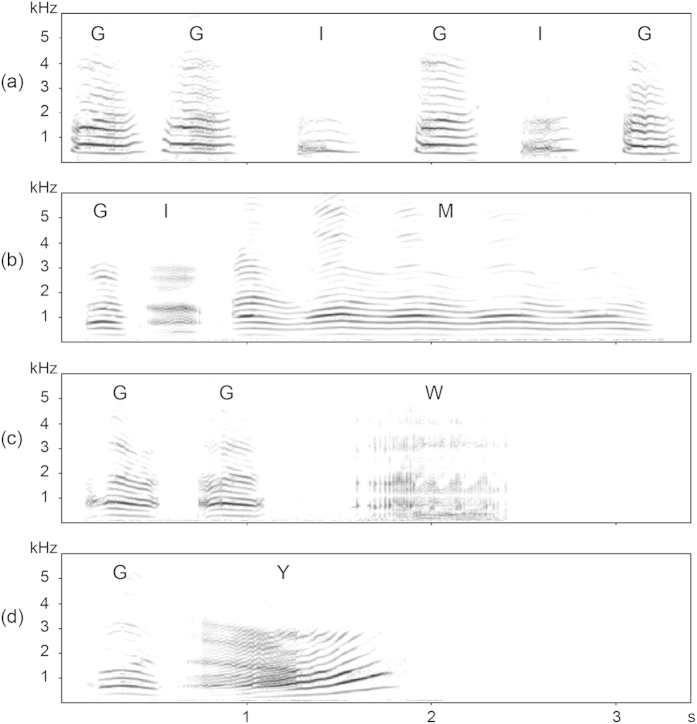
Example playback stimuli of a (a) grunt-only vocal sequence and derived call vocal sequences that include either a (b) moan, (c) wobble, or (d) yawn. ‘G’ refers to exhaled grunts, ‘I’ refers to inhaled grunts, ‘M’ refers to a moan, ‘W’ refers to a wobble, and ‘Y’ refers to a yawn. All of the vocal sequences include exhaled and inhaled call types. Spectrograms were made with Avisoft SAS Lab Pro.

**Figure 2:**
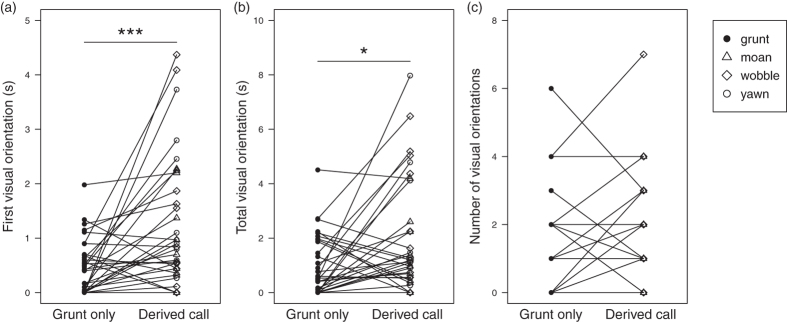
Visual orientation towards the speaker following the conclusion of unfamiliar male vocal sequences. Vocal sequences were composed of only grunts (grunt) or included one of the derived calls (moan, wobble, yawn). Behaviors measured included (**a**) the duration of the first visual orientation towards the speaker, (**b**) the duration of the total visual orientation towards the speaker, and (**c**) the number of distinct visual orientations towards the speaker. Lines connect trials carried out with the same female subject. *p < 0.05, **p < 0.01, ***p < 0.001.

There was good evidence that female geladas distinguished between simulated sequences of grunt only and derived calls from unfamiliar males (Fig. 1). The first visual orientation that females made towards the speaker in the minute following the conclusion of playback stimuli was longer following derived call sequences (mean ± SE [range]: 1.139 s ± 0.190 [0.000–4.370 s]) than to grunt only sequences (0.421 s ± 0.070 [0.000–1.980 s]; W = 73.5, N = 36, p = 0.0007; Fig. 2a). Females also spent more time overall visually orienting towards simulated sequences of derived calls (1.834 s ± 0.305 [0.000–7.970 s]) than to grunt only sequences (0.873 s ± 0.145 [0.000–4.510 s]; W = 134, N = 36, p = 0.0261; Fig. 2b). There was no evidence that females made different numbers of separate visual orientations towards the speaker following simulated derived call (1.583 looks ± 0.264 [0–7 looks]) and grunt only sequences (1.417 looks ± 0.236 [0–6 looks]; W = 113, N = 36, p = 0.6669; Fig. 2c). Visual orientation towards the three types of derived call sequences were similar, although on average, females tended to looked longer towards sequences including wobbles or yawns than those including moans (Table 1).

There was no evidence that the presentation order of playback stimuli affected females’ visual orientation responses to vocal sequences from unfamiliar males. The first visual orientation that females made towards the speaker in the minute following the playback stimuli was similar following the vocal sequence of the first playback trial (0.913 s ± 0.152 [0.000–4.089 s]) compared to the second playback trial (0.648 s ± 0.108 [0.000–4.370 s; W = 322.5, N = 36, p = 0.1470]). Females also spent a similar amount of time overall visually orienting towards the vocal sequence of the first playback trial (1.524 s ± 0.254 [0.000–6.482 s]) as to the second playback trial (1.183 s ± 0.197 [0.000–7.970 s]; W = 305, N = 36, p = 0.2682). Additionally, females made a similar number of distinct visual orientations (i.e., “looks”) towards the speaker following the vocal sequence of the first playback trial (1.639 looks ± 0.273 [0–7 looks]) as to the second playback trial (1.361 looks ± 0.227 [0–4 looks]; W = 163.5, N = 36, p = 0.2270).

### Time spent in proximity to the speaker

**Figure 3:**
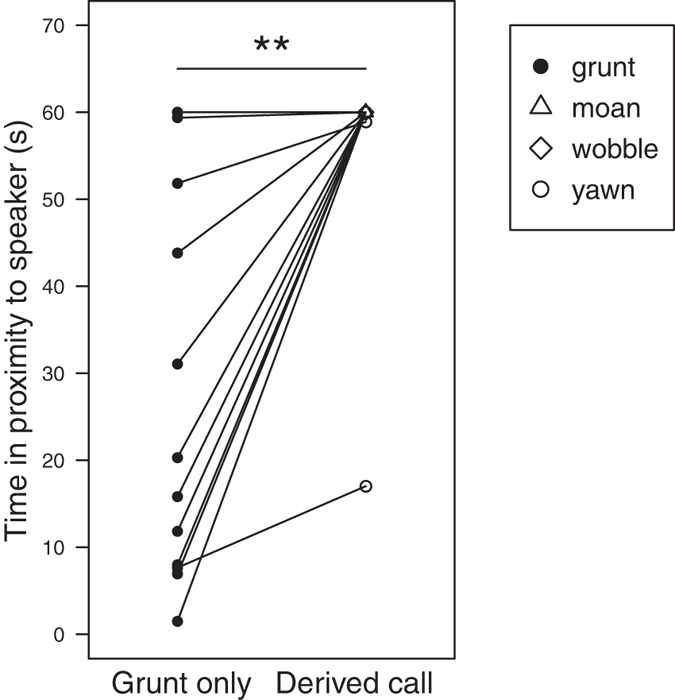
Time spent in proximity to the speaker following the conclusion of unfamiliar male vocal sequences. Vocal sequences were composed of only grunts (grunt) or included one of the derived calls (moan, wobble, yawn). Lines connect trials carried out with the same female subject. *p < 0.05, **p < 0.01, ***p < 0.001.

There was evidence to indicate that females spent more time in proximity to the speaker following simulated derived call sequences (58.775 s ± 9.796 [17.027–60.000 s]) than to grunt only sequences (48.836 s ± 8.139 [1.501–60.000 s]; W = 0, N = 36, p = 0.0039; Fig. 3). Proximity behavior was the same towards the three types of derived call playback stimuli in that females almost always spent at least one minute in close proximity to the speaker upon hearing a moan, wobble, or yawn sequence (Table 1). There was no evidence that the presentation order of playback stimuli affected the amount of time that females spent in proximity to the speaker. Females spent a similar amount of time in proximity to the speaker following the vocal sequence of the first playback trial (56.139 s ± 9.357 [11.820–60.000 s]) as to the second playback trial (51.472 s ± 8.579 [1.501–60.000 s]; W = 50, N = 36, p = 0.1424).

## Discussion

This is the first study to systematically show that vocal complexity may be driven by inter-sexual selection in a non-human primate. We found that female geladas clearly distinguished between derived and homologous calls, and the direction of the differences in responding all suggest a stronger salience of and, possibly a preference for, the derived calls. Specifically, females hearing playbacks of male vocal sequences containing one of three derived call types – moans, wobbles, and yawns – oriented longer as well as spent more time in proximity to the speaker. These results align with an extensive body of research in songbirds demonstrating that diversified male vocal signals may function to attract mates and establish long-term social bonds^16^,^17^,^18^,^19^,^20^,^21^.

Although similar evidence in mammalian species is relatively sparse^30^, our results do support a growing body of work in rodents and bats suggesting that vocal complexity may have analogous social functions in mammals. As with female geladas, female mice (*Mus musculus*) spend more time around playback stimuli of male song containing many elaborate syllable types than those composed of only a simple syllable type^41^. In addition, male greater sac-winged bats (*Saccopteryx bilineata*) producing songs with several unique syllable types have more females who consistently roost in their harem territories than do males producing fewer syllable types^45^. Therefore, both male geladas and sac-winged bats utilizing a a more complex string of sounds may be better equipped to maintain bonds with the females in their harem-like reproductive units. Thus, these results are the first to indicate that the diversification of call types may have evolved as a male-specific strategy to maintain long-term social bonds with females in primates.

Our findings also have implications for gelada society and the evolution of tolerance. Females had generally weak responses and stayed in proximity to derived call sequences (and many of the grunt sequences) even though the males vocalizing were unfamiliar to the subject. This is surprising considering that the close and sudden presence of a stranger is a rare and potentially distressing event for primates that tend to live in long-term and relatively stable social groups^46^. One likely correlate of this apparent tolerance of strangers is that females may simply be unable to recognize the vocal signals of males from their band but outside of their reproductive units and are consequently quite habituated to hearing calls from unrecognized individuals. This corresponds with previous evidence showing that male geladas do not distinguish vocal sequences of familiar males from unfamiliar males^47^. Another possibility is that, in addition to a primary function of derived calls in male-female bonding within units, the calls may have an inherent attractiveness that leads to a secondary function in maintaining cohesion across units. Geladas live in fission-fusion societies, and it is a regular occurrence for reproductive units to travel with unfamiliar units^37^. Derived calls may play a particularly important role in coordinating these flexible group dynamics. Additionally, female composition in the reproductive units is stable, but leader male tenures rarely last more than a few years^39^. This means that females may need to rapidly form strong associations with new leader males following takeovers. An intriguing line of future research will be to explore how new leader males may use derived calls as a strategy to develop their social bonds with females.

It is still unknown whether females are attending specifically to ‘derived calls’ or to ‘complex vocal sequences’. Orientation responses are notoriously difficult to interpret^43^, and so derived calls may invoke a greater orientation response than grunts because they are rarer or more indicative of salient social stimuli instead of reflecting female interest^28^. However, females stayed close to simulated sequences of derived calls, suggesting that it is not simply the case that those calls are startling. Also, given that geladas live in large fission-fusion societies and vocalize at a high rate^28^, it is not unusual for females to hear complex vocal sequences from unfamiliar males. Instead, our findings indicate that females may show a preference for innovative vocal signals, which would align with studies of non-primate taxa like zebra finches (*Taeniopygia guttata*)^19^. Derived calls also are characterized by acoustic properties that may make them more elaborate and potentially more attractive than the typical grunt (e.g., long duration, frequency modulation and large bandwidth). Such acoustic properties are also found in call types preferred by female birds^22^,^23^,^48^,^49^, anurans^50^, and other mammals^41^,^45^,^51^. Conversely, female geladas may be attending to the degree of complexity in vocal sequences rather than to the specific use of derived calls. This explanation would align with studies showing that female birds are attracted to vocal stimuli containing large syllable and song repertoires^16^,^17^,^18^,^19^,^20^,^21^. Even in many of these bird studies, however, it is unclear whether females are exhibiting a preference for large repertoires or whether repertoire diversification is actually driven by a greater signal value in individual components^14^. In both birds and geladas, it is difficult to untangle these explanations because presenting females with individual components alone rather than in sequences would be an unnatural stimulus that may evoke responses that are not ecologically relevant.

Future studies are needed to tease apart the types of information gained from different vocal sequence combinations made by geladas. Females in our study did not appear to distinguish between sequences containing different derived call types, but small sample sizes prevented us from making statistical comparisons. One exciting possibility is that derived call vocal sequences are honest indicators of mate quality (being produced at a higher rate by the best quality males), which would make them particularly attractive to females^52^. Given that every male used in this study contributed equal numbers of derived call and grunt sequences, it is unlikely that our results reflect female preference for the acoustic qualities of specific males^42^. Instead, it seems more likely that gelada females prefer males giving a higher output of derived call vocal sequences. Further work will be needed to test the possibility that variation in the complexity of male vocal behavior translates to reproductive success. Until then, we are unable to completely rule out the possibility that variation in female orientation and proximity behavior in response to male calls reflect differences in motivation to engage with a *social* partner rather than a *sexual* partner. Additionally, these derived call sequences may serve an alternative or complimentary role in female detection and identification of unit males^53^. In other words, derived call sequences could counteract environmental noise such as the chorus of other geladas in their large communities. Similar solutions to cope with conspecific noise has been proposed for other species such as Túngara frogs (*Physalaemus pustulosus*)^50^.

There is great debate over the evolutionary origins of highly complex and diversified forms of communication such as human language. One focus has been on investigating comparative evidence for semantic communication as a key driver in the evolution of complex communication^54^,^55^. Despite the small vocal repertoires of non-human primates, there are many well-studied examples of how diversified primate calls and call combinations may have evolved as a tool to communicate functionally referential information about food and predators^56^,^57^,^58^. Another focus has been on investigating comparative evidence for elaboration in affective communication or types of holistic communication that may seem multi-faceted in appearance but serve singular social functions^59^. While best exemplified by the elaborate bird songs that function to successfully interact with mates and deter rivals^15^, there is only limited evidence that non-human primate vocal systems can be elaborate in similar contexts (e.g., gibbons (*Hylobates sp.*)^60^). By providing novel evidence of a potential role for sexual selection in the emergence of vocal complexity in primate vocalizations, our results build support for non-semantic vocal elaboration as an early step towards language^34^.

## Methods

### Study site and animals

Experimental playback data were collected from February to June 2014 in the Sankaber area of Simien Mountains National Park, Ethiopia. Research was approved by the University Committee on Use and Care of Animals (UCUCA) at the University of Michigan and was carried out in accordance with the laws and approved guidelines of Ethiopia. Study subjects were 36 adult female geladas from outside the three main study bands followed by the University of Michigan Gelada Research Project since 2006. Females from outside of the main study bands were chosen so they would be uniformly unfamiliar with the males from which we recorded playback stimuli. Although the vocalizations used here are typically exchanged between familiar individuals, it is very difficult to conduct realistic playback trials among members of the same unit as they are usually within visual contact of each other. In addition to being more tractable, using unfamiliar callers and subjects also controls for variation in relationship quality between caller and subject, ensuring that any differences in responses are likely due to differences in the stimuli. Female subjects were habituated to humans on foot up to 3–5 m and could be identified by unique body markings (e.g., ear tears and coloration).

### Playback stimuli and experimental design

Vocal sequences were recorded from 12 adult male geladas who were unit leaders and/or followers from one of the three main study bands between 2008–2014. These recordings were made from less than 10 m using a Sennheiser ME-66 directional microphone and a Marantz PMD 660 or 661 digital recorder. Playback stimuli were made using PRAAT 5.2.29 for Macintosh. Each playback stimulus was composed of 2–9 calls from a natural sequence with a high signal-to-noise ratio. The majority of recorded vocal sequences were manipulated (e.g., excluding call(s) from the beginning and/or end of a sequence) to produce playback stimuli that were clear of overlapping calls and were of a similar overall duration. The amplitude of extraneous sounds (e.g. bird chirp or vocalization from another gelada) found in the intervals between calls was dampened using PRAAT.

The playback stimuli consisted of 18 ‘grunt only’ and 18 ‘derived call’ vocal sequences. Grunt only sequences were composed of exhaled grunts and inhaled grunts (mean ± SE [range]: 5.000 ± 0.406 [3–9] calls per stimulus). Derived call vocal sequences (3.222 ± 0.4759 [2–9] calls per stimulus) were composed of exhaled and inhaled grunts mixed with one of the three elaborate derived calls: exhaled moans (n = 6 stimuli; 2.500 ± 1.021 [2–4] calls per sequence), exhaled (n = 3) or inhaled (n = 3) wobbles (4.500 ± 1.837 [2–9] calls per sequence), and inhaled vocalized yawns (n = 6; 2.667 ± 1.089 [2–4] calls per sequence). The elaborate derived calls were acoustically different from exhaled and inhaled grunts in various ways: exhaled moans, wobbles, and yawns used in the derived call playback stimuli were longer in duration compared to exhaled and inhaled grunts, wobbles had the highest frequency modulation, and yawns had the highest formant (F1) frequency (Table S1). For further descriptions of these call types, see Gustison *et al*.^28^, and see Fig. 1 for spectrograms of grunt only and derived call sequence playback stimuli.

Six study males contributed one grunt only and one derived call sequence and six study males contributed two grunt only and two derived call sequences. Each of the grunt only sequences was paired with a derived call sequence – forming 18 playback ‘sets’. A counterbalanced matched-control design was used; 18 of the study females were presented with a grunt only sequence first and a derived call sequence second, and the other 18 females were presented with a derived call sequence first and a grunt only sequence second. Therefore, the 36 female subjects were each exposed to two playback stimuli (grunt only and derived call vocal sequences) for a total of 72 playback trials. This repeated measure design ensured that the variation in internal (e.g., reproductive state) and external (e.g., recent interactions with unit members) factors were similar for female subjects exposed to grunt only and derived call simulated sequences.

Several precautions were taken to ensure that playback stimuli of grunt only and derived call sequences were similar except for the call composition. First, we controlled for other acoustic signals that could affect female responses, like inter-male variation in fundamental frequency, by matching male callers across playback sets. For every playback set that consisted of a grunt only sequence from male A and a derived call sequence from male B, there was a corresponding playback set that consisted of a grunt only sequence from male B and a derived call sequence from male A. Second, variation in female response due to vocal sequence duration was controlled for by matching the durations of grunt only (2.768 s ± 0.652 [1.411–3.782]) and derived call (2.637 s ± 0.621 [1.698–3.680]) vocal sequences in a playback set (Wilcoxon signed-rank test: W = 123, N = 18, p = 0.1084). Vocal sequence duration also was similar across the three types of derived call sequences (Mann-Whitney U tests: moan (3.031 s ± 1.237 [2.264–3.680]) vs. wobble (2.447 s ± 0.999 [1.945–3.170]): U = 29, N1 = N2 = 6, p = 0.0931; moan vs. yawn (2.432 s ± 0.993 [1.698–3.361]): U = 28, N1 = N2 = 6, p = 0.132; wobble vs. yawn: U = 20, N1 = N2 = 6, p = 0.8182). Third, grunt only and derived call sequences were played at a similar decibel level. A Radioshack Digital Sound Level Meter was used to check that the maximum dB of each sequence in a quiet indoor environment was similar for grunt only (69.833 dB ± 16.460 [67–75]) and derived call sequences (69.667 dB ± 16.421 [64–78]; Wilcoxon signed-rank test: W = 57.5, N = 18, p = 0.7764). The maximum dB also was similar across types of derived call sequences (Mann-Whitney U tests: moan (68.167 dB ± 27.829 [64–72]) vs. wobble (70.667 dB ± 28.850 [67–78]): U = 11.5, N1 = N2 = 6, p = 0.3315; moan vs. yawn (70.167 dB ± 28.645 [67–74]): U = 10.5, N1 = N2 = 6, p = 0.2573; wobble vs. yawn: U = 15.5, N1 = N2 = 6, p = 0.7462).

### Playback protocol

An adult female was chosen as a subject for a playback trial if she was relatively stationary (i.e., feeding or resting), was not engaged in social activity, and was close to vegetation where the speaker could be hidden. No individuals were located between the subject and the speaker. During playback sessions, playback stimuli were presented with a SanDisk Clip mp3 player connected to a Bose Roommate II portable loudspeaker. The speaker was concealed behind vegetation 3–10 m from the subject in the direction of the gelada band. Thus, playback stimuli presumably represented vocal sequences from unit males rather than bachelors^47^. Female behavior was recorded with a Kodak PlaySport HD waterproof pocket video camera. The playback stimulus was played after 10 s of video recording if the subject remained engaged in non-social stationary behavior and her body and head were oriented 90 degrees from the speaker. The subject continued to be video recorded for one minute following the playback stimulus. Previous research has shown that one minute adequately captures gelada responses to contact calls such as grunts^47^. The second trial involving the same subject was played at least two minutes after the end of recording the first trial from a location that was at least 3 m away from the location of the first playback stimulus. At the end of each playback set, the relative locations and distances between the video recorder, closest adult unit male, speaker and study female (at both the presentation of the playback stimuli and after one minute) were recorded. The presentation order of different playback sets was randomized across subjects.

Following the guidelines put forth by Fischer *et al*.^43^, several precautions were made to avoid habituation of female subjects to the playback stimuli. For example, we played male vocal sequences at a much lower rate than they occur naturally; we played no more than three playback sets per day (6 vocal sequences), whereas gelada males naturally produce vocal sequences including grunts and derived calls at least 14 times per hour on average^28^. Given that geladas range in bands that include many males, females are exposed to these vocal sequences at a much higher rate^28^. For playback sets carried out on the same day, we chose female subjects from different units that were out of sight (and likely audible range) from the location of the previous playback set. Playback stimuli were never repeated on the same day.

### Analysis of female behavioral responses to playback stimuli

An independent observer scored behavior using Windows Live Movie Maker 2011 (Microsoft, Redmond, WA, USA) from at least 10 seconds before to over one minute after the playback stimulus using frame-by-frame analysis (behavioral responses were later calculated from the time frame from the end of the playback stimuli to one minute after). The sound was turned off so that the observer was blind to when and what type of playback stimulus was played. The observer scored the start and end of all visual orientation towards the speaker, defined as the subject’s head being oriented ±45 degrees in the direction of the speaker. From these data, we computed three specific visual orientation measures: duration of the first visual orientation towards the speaker, duration of the total visual orientation towards the speaker, and the number of separate visual orientations towards the speaker. All behavioral measures were taken from the end of the playback sequence so that it could be assured that we were quantifying the female responses to an entire sequence. Because of this, we did not measure a female’s latency to look, which is common in playback experiments with non-human primates^43^. Females often looked towards the speaker prior to the conclusion of the sequence, and so “lag to look” is not a relevant measure of female response to the entire playback stimulus. The independent observer also scored the videos for the amount of time that the female spent in proximity to the speaker, defined as the time post-stimulus until the female moved over 1 m in a direction 90–180 degrees from the speaker. We focus on proximity behavior rather than other potential ‘preference’ behaviors such as approach or copulation displays because the social structure of wild geladas is such that females do not commonly engage in close-range affiliative interactions (e.g., approach, grooming, and copulation) with non-unit males. Previous studies suggest that female geladas do not mate with non-unit males, and if they mate with subordinate follower males in their units, they are at risk of aggression from the leader male^38^,^39^. In the present study, there was only one instance following a grunt only sequence that the female subject moved closer to the speaker.

To check for intra-observer reliability, the observer re-scored each video for a second time at least 56 days after the original scoring. Rho values from Spearman signed-rank correlations were used to assess consistency in the four analyzed behaviors. All behaviors had intra-observer reliability rho values over 0.84 (duration of first visual orientation −0.877; duration of total visual orientation −0.876; number of looks −0.863; time spent in proximity to speaker −0.840).

We used Shapiro-Wilk tests to check whether behavioral variables fit a normal distribution. No variables fit a normal distribution, and this remained true after transformation (square-root transformation, p < 0.05; natural log transformation, p < 0.05). Therefore, we used non-parametric tests. For each behavioral measure, we first used Wilcoxon sign-rank tests to see if females responded differentially to grunt only and derived call vocal sequences. We did not compare responses to different types of derived calls because of small sample sizes. Second, we used Wilcoxon sign-rank tests to see if there were any order effects by comparing within-female responses to the first and second playback stimulus. All tests were two-tailed (α = 0.05) and carried out using Cran R package version 3.1.1. Descriptive statistics are reported as mean ± SE [range].

## Additional Information

**How to cite this article**: Gustison, M. L. and Bergman, T. J. Vocal complexity influences female responses to gelada male calls. *Sci. Rep.* **6**, 19680; doi: 10.1038/srep19680 (2016).

## Supplementary Material

Supplementary Information

## Figures and Tables

**Table 1:** Results for visual orientation and proximity responses to the three types of derived call playback stimuli.

**Dependent variable**	**N trials**	**Mean ± SE (s)**	**Range (s)**
*First visual orientation*			
Moan	12	0.905 ± 0.261	0.000–2.270
Wobble	12	1.333 ± 0.385	0.000–4.370
Yawn	12	1.179 ± 0.340	0.000–3.730
*Total visual orientation*			
Moan	12	1.172 ± 0.338	0.000–4.190
Wobble	12	2.394 ± 0.691	0.000–6.482
Yawn	12	1.937 ± 0.559	0.000–7.970
*Number of visual orientations*			
Moan	12	1.250 ± 0.361	0–4
Wobble	12	2.167 ± 0.625	0–7
Yawn	12	1.333 ± 0.385	0–3
*Time spent in proximity to speaker*			
Moan	12	60.000^a^	60.000^a^
Wobble	12	60.000^a^	60.000^a^
Yawn	12	56.326 ± 16.260	17.027–60.000

^a^All subjects engaged in this behavior for at least 60 seconds.
