# Supplementary material for: Vocal complexity influences female responses to gelada male calls
Source: Sci Rep. 2016 Jan 21;6:19680. doi: 10.1038/srep19680 (PMC4726186; doi:10.1038/srep19680)
Supplement: Supplementary Information [file srep19680-s1.pdf]

Title: Vocal complexity influences female responses to gelada male calls

Author Information: Morgan L. Gustison and Thore J. Bergman

Supplementary Table 1

Table S1. Acoustic parameters for call types used in playback stimuli, reported in mean ± SE [range].

Call type	N	Duration (s)[a]	F1 Frequency (Hz)[b]	F1 Bandwidth (Hz)	F1 Modulation (CV)
Exhaled grunt	94	0.324 ± 0.012 [0.136 – 0.766]	714.168 ± 8.084 [515.224 – 859.491]	421.950 ± 27.350 [58.464 – 1202.765]	12.988 ± 0.798 [2.336 – 34.448]
Inhaled grunt	30	0.470 ± 0.068 [0.101 – 1.430]	764.651 ± 24.096 [523.135 – 968.694]	586.732 ± 67.626 [82.359 – 1414.826]	16.350 ± 2.011 [2.592 – 47.127]
Moan	6	2.131 ± 0.274 [1.265 – 3.247]	746.734 ± 37.475 [593.810 – 857.523]	417.137 ± 69.587 [149.807 – 623.556]	9.019 ± 1.066 [5.757 – 12.319]
Wobble	6	1.129 ± 0.192 [0.507 – 1.680]	750.921 ± 38.804 [631.204 – 871.575]	707.137 ± 193.463 [227.722 – 1504.683]	19.822 ± 6.110 [7.709 – 44.466]
Yawn	6	1.321 ± 0.166 [0.728 – 1.840]	817.020 ± 29.881 [710.988 – 886.753]	619.122 ± 100.842 [333.326 – 987.294]	14.900 ± 2.438 [8.046 – 22.304]

[a] Duration calculated with Avisoft SASLab Pro.
[b] The 1st formant (F1) was calculated with PRATT using standard settings. Coefficients of variation (CV) were calculated from the listing of all first formant (F1) measures (every 0.00625 s increment) for individual calls. Fundamental frequency (F0) was not included because it could not be determined for all calls.
